# Adaptive learning and recall of motor-sensory sequences in adult echolocating bats

**DOI:** 10.1186/s12915-021-01099-w

**Published:** 2021-08-19

**Authors:** Mor Taub, Yossi Yovel

**Affiliations:** 1 Department of Zoology, Faculty of Life Sciences, Tel Aviv University, 6997801 Tel Aviv, Israel; 2 Sagol School of Neuroscience, Tel Aviv University, 6997801 Tel Aviv, Israel

**Keywords:** Bats, Echolocation, Sensory acquisition, Sensory planning, Adaptive-learning

## Abstract

**Background:**

Learning to adapt to changes in the environment is highly beneficial. This is especially true for echolocating bats that forage in diverse environments, moving between open spaces to highly complex ones. Bats are known for their ability to rapidly adjust their sensing according to auditory information gathered from the environment within milliseconds but can they also benefit from longer adaptive processes? In this study, we examined adult bats’ ability to slowly adapt their sensing strategy to a new type of environment they have never experienced for such long durations, and to then maintain this learned echolocation strategy over time.

**Results:**

We show that over a period of weeks, *Pipistrellus kuhlii* bats gradually adapt their pre-takeoff echolocation sequence when moved to a constantly cluttered environment. After adopting this improved strategy, the bats retained an ability to instantaneously use it when placed back in a similarly cluttered environment, even after spending many months in a significantly less cluttered environment.

**Conclusions:**

We demonstrate long-term adaptive flexibility in sensory acquisition in adult animals. Our study also gives further insight into the importance of sensory planning in the initiation of a precise sensorimotor behavior such as approaching for landing.

**Supplementary Information:**

The online version contains supplementary material available at 10.1186/s12915-021-01099-w.

## Background

Acquiring sensory information is crucial for all organisms. Proper sensing depends on multiple processes, such as filtering sensory information, sensory adaptations (e.g., pupil dilation), and active sensing (e.g., eye movements). The ontogeny of these processes is far from understood both at the behavioral and at the neural levels. Specifically, we know little about which processes have to be learned and whether they are plastic during life. Several previous studies have shown sensory plasticity in the neural encoding of the sensory environment across sensory modalities in various species including humans [1–7], ferrets [8], owls [9], and monkeys [10].

Far fewer examples exist that demonstrate adult adaptive flexibility in active sensing. Humans, for example, depend on eye movements to visually sense the world [11–15]. Humans are known to move their eyes differently depending on the task [15], but it is not known if they can learn a new eye movement strategy that is beneficial for a task they have never encountered before. This is what we tested in echolocating bats.

Echolocating bats are famous for their ability to adjust sensory acquisition according to their environment [16–27]. Some bat species routinely and rapidly move between environments that differ in their echoic-complexity and their level of echo-reflectivity. The degree of environmental background echoes is termed clutter and is arguably the most important parameter driving adjustments in echolocation. Bats have mostly been shown to adjust their echolocation within their common ecological niche, but the extent to which they can modify their echolocation strategy to other environmental contexts remains unclear. Such adaptive learning could be ecologically relevant when foraging in a novel previously un-encountered environment. For example, can a bat that is used to hunt above meadows where clutter is rather low expand its niche to hunt in a highly cluttered forest and, accordingly, adapt its echolocation to this new environment over-time. *Pipistrellus kuhlii* bats, the species we focus on in this study, often reside in small crevasses inside buildings, which require maneuvering through high clutter, and they then fly out to forage in areas ranging from mildly dense vegetation to open spaces. Such rapid changes in clutter require quick adjustments of sensory acquisition, and indeed, previous studies found a wide range of flexibility in the echolocation of *P*. *kuhlii* and other bats [25, 27–31]. Notably, these rapid sensory adjustments are performed within milliseconds from sensing an environmental change such as an increase in clutter. In this study, we tested the hypothesis that in addition to rapid changes, bats can also gradually adapt, over time, in response to a new unfamiliar environment.

More specifically, we aimed to examine whether adult *P. kuhlii* bats can adapt their sensing to a new constantly cluttered environment, probably more cluttered than they have ever experienced for long periods. We further aimed to test whether such adaptations will be permanently learned by the bats, allowing them to apply the appropriate echolocation strategy when faced with similar cluttered environments later on in the future. To this end, we placed the bats in a constantly cluttered flight chamber and recorded their echolocation over time, documenting its adaptation. The echolocation sequence has been previously described as containing sonar (strobe) groups, which have been suggested to play an important role in the fine tuning of auditory scene analysis [32]. Here we focus on the intervals between two such groups, in the echolocation sequence emitted right before take-off (which we term the inter-group interval-IGI). We found that over the course of 2 months, the bats continuously decreased these pre-takeoff intervals, gradually broadening their echolocation repertoire. These adaptations take relatively more time than the previously described rapid adjustments. Furthermore, after 6 months in a less cluttered environment, once re-introduced into a constantly cluttered environment, the bats immediately started using the suitable adapted echolocation strategy, suggesting adaptive learning in sensory acquisition.

## Results

**Fig. 1:**
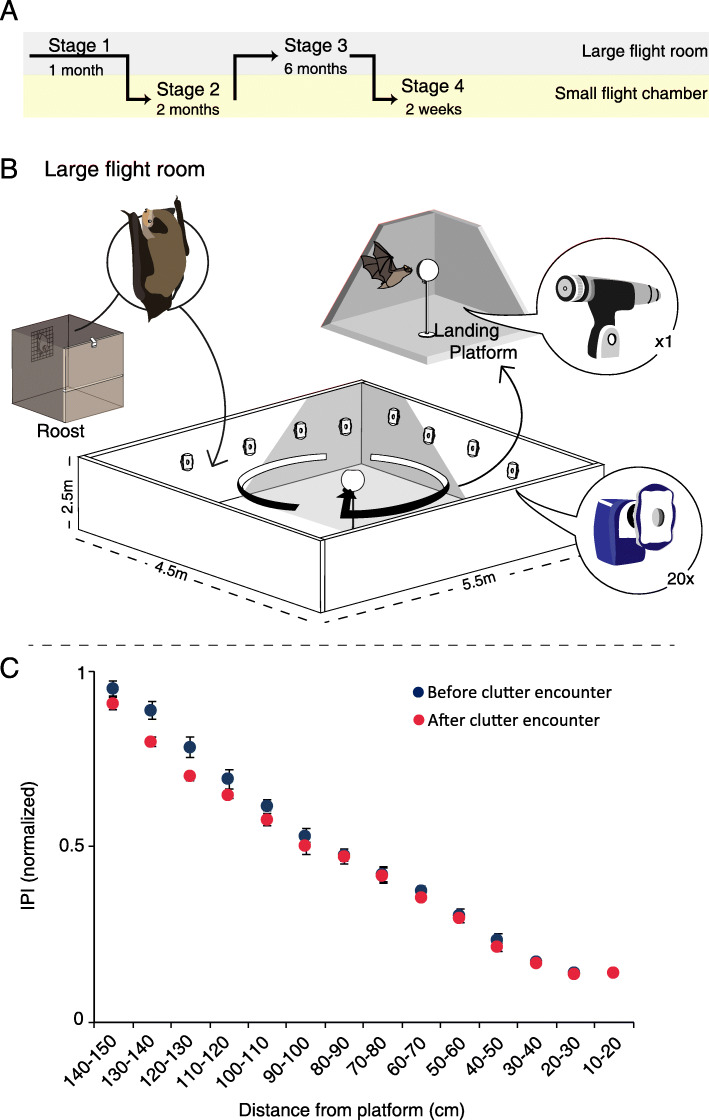
Inter-pulse interval (IPI) in the large flight room. **A** The experiment consisted of four stages, moving between two environments: a large flight room (grey) and a smaller cluttered flight chamber (yellow). **B** The large flight room was 5.5 × 4.5 × 2.5 m^3^. The landing platform was located in the center of the room with an ultrasonic microphone attached to it and directed towards the bat. Twenty tracking cameras recorded the flight path of the bat. **C** The IPI measured during the approach phase of the echolocation in the last 150 cm before landing for two time points in the experiment (mean ± SE; *n* = 5): before the cluttered chamber experiment (stage 1), and immediately after the cluttered chamber (stage 3). SE’s are depicted, but are very small. Data was normalized for all individual bats by dividing each bat’s data by its maximum value (the maximum across all trials)

**Fig. 2:**
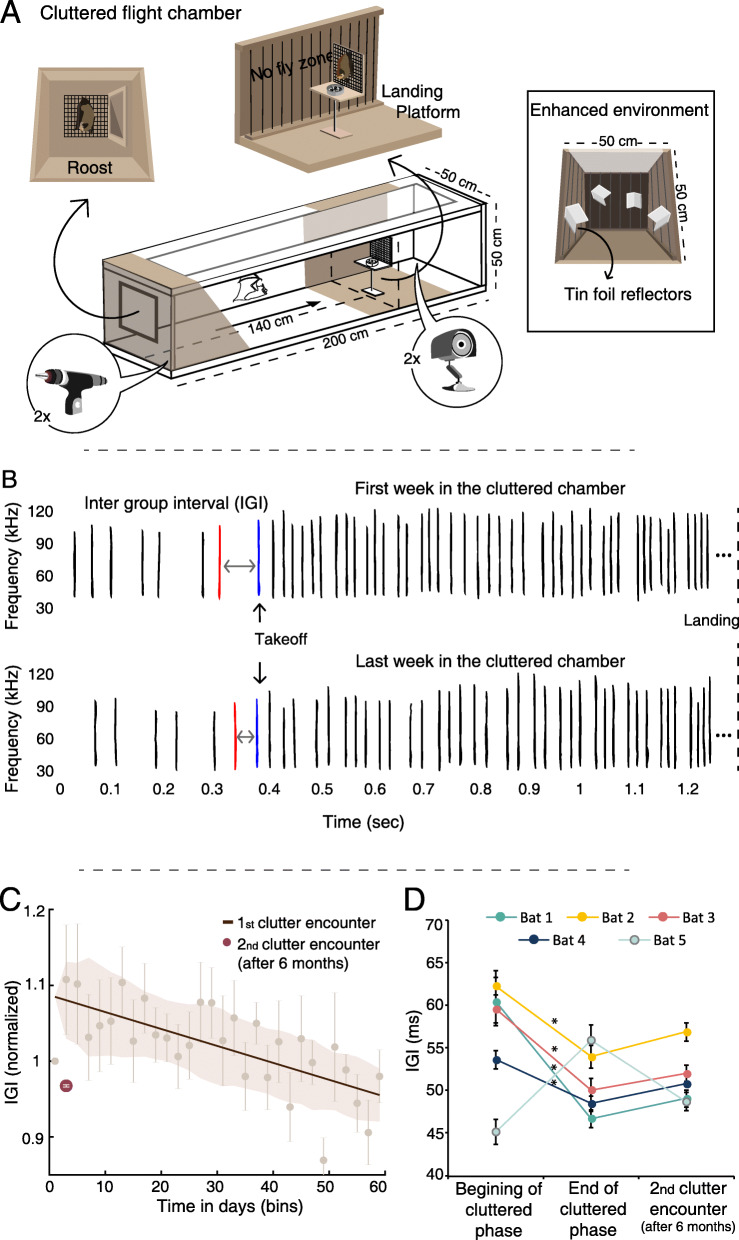
Bats adjust sensory acquisition in time in a constantly cluttered environment. **A** The smaller cluttered flight chamber was 200 × 50 × 50 cm^3^. Two ultrasonic microphones and two surveillance cameras recorded the bats. The landing platform was located 140 cm from the roost wall at the far end of the chamber. In the fourth stage of the experiment, ten tin foil reflectors were added to the chamber in order to change the acoustic complexity (enhanced environment). **B** A typical echolocation sequence of the approach phase from the start of the first encounter (top) and the end (bottom). The inter-group interval is defined as the time between the start of the last pulse before takeoff (red) and the first pulse after take-off (blue). **C** There was a decrease in IGI over the course of 2 months (brown line depicts the linear fit and shading shows SE; points show mean ± SE, n = 5 bats). On their return to the chamber after 6 months, the bats immediately used shorter IGI values (2nd clutter encounter, bourdeaux circle). Data was normalized for all individual bats by dividing each bat’s data by the average value of its first day. **D** IGI values of the five individual bats at three different time points along the experiment: the first 2 weeks in the chamber (beginning of cluttered phase), the last 2 weeks in the chamber (end of cluttered phase), and 2 weeks in the chamber after 6 months in the large flight room (2nd clutter encounter). There was a significant decrease in IGI between the start and end of the first encounter (mean ± SE) but not between the end of the first encounter and the second encounter (for four out of the five bats). Asterisk indicates a significant change in the same direction as the group

Five *Pipistrellus kuhlii* bats were trained to land on a platform in two different environments (Fig. 1A and Additional file 1: Figure S1). In the first stage of the experiment, the bats were flown individually, for 3 days, in the large flight room (5.5 × 4.5 × 2.5 m^3^, Fig. 1B) and their echolocation during the last 150 cm of flight before landing was analyzed (i.e., during the approach phase; Fig. 1C and Additional file 2: Figure S2: before clutter encounter). The bats were then transferred into a very small, constantly cluttered flight chamber (200 × 50 × 50 cm^3^, Fig. 2A) where they spent their entire time while we recorded their echolocation for 2 months from their first landing (stage 2). The bats learned to fly to and land on a platform located 140 cm from their roost where mealworms were offered. All other walls were restricted with nylon wire, forcing the bats to initiate flight only from the roost (Fig. 2A). We continuously recorded their echolocation and analyzed several echolocation parameters including the interval before the last two (strobe) groups just before take-off (the IGI, see Fig. 2B). We focused on this parameter, because we previously found that bats will adjust it according to the distance of the target they are about to fly to [27]. In this previous study, we found that in such cluttered environments, bats assess the distance of the target using echolocation before take-off, shortening the inter-group-interval when the target is closer [27]. Over their 2 months in this environment, the bats significantly and continuously reduced their IGI, right before takeoff (Fig. 2C, mixed-effect generalized linear model (GLM) with the IGI set as the explained factor, time as a fixed factor and bat ID as a random effect, Bonferroni corrected *P* < 0.0005, *F* = 17, df = 1, *n* = 5). The result was consistent in the individual level—with four of the five individuals showing a significant decrease in IGI (Additional file 3: Figure S3, See Additional file 4: Table S1 for individual statistics). Bat 5 was an outlier and showed opposite patterns in most echolocation parameters. In accordance with the reduction in emission intervals, there was also a significant decrease in pulse intensity over time (mixed-effect GLM as above, Bonferroni corrected *P* = 0.05, *F* = 6.8, df = 1; Additional file 5: Figure S4) but only two individuals significantly reduced calling intensity (See Additional file 4: Table S1 for individual statistics). Notably, these changes in echolocation are in-line with the direction of the adjustments that are well documented in a cluttered environment, i.e., shortening the intervals between pulses and decreasing their intensity [19, 33], but such adjustments typically occur within milliseconds. Echolocating bats are known to adjust their echolocation to clutter immediately, while here we report continuous gradual adjustments that required weeks, longer than anything previously reported, suggesting that the bats were adapting their sensing strategy over time. There was no significant change in peak frequency (mixed-effect GLM as above, Bonferroni corrected *P* = 0.3, *F* = 3.7, df = 1, *n* = 5 bats), and there was also no significant change in pulse duration during this time (mixed-effect GLM as above, *P* = 0.5, *F* = 2.5, df = 1, *n* = 5 bats), but we should note that pulse duration is often a parameter that is more difficult to measure (especially when measuring such short pulses).

The analyses above were all for the echolocation before take-off. To test whether bats also adapted their echolocation during flight, e.g., by reducing inter-pulse intervals to increase information update-rate, we examined the number of pulses emitted during the flights (from take-off to landing). There was no significant change in the number of pulses emitted throughout the 2 months (Additional file 6: Figure S5, Mixed effect GLM as above, Bonferroni corrected *P* = 0.15, *F* = 0.5, df = 1, *n* = 5). A few individuals changed their calling rate, but in different directions (see Additional file 4: Table S1 for statistics).

After 2 months in the cluttered flight chamber, the bats were returned to the large flight room and were recorded again for 4–8 days (Fig. 1C, after clutter encounter). Overall, on their return to the large room, some of the bats used significantly different inter-pulse-intervals (IPI) than before entering the cluttered environment (repeated measures ANCOVA, *P* = 0.02, *F* = 3.8, df = 2, *n* = 5 bats). However, the bats did not exhibit a consistent change: three bats significantly reduced the IPI at this stage while one bat significantly increased and one did not change (Additional file 2: Figure S2 and Additional file 4: Table S1). These inconsistent changes could have been a response to the clutter experienced in the previous 2 months, but they could have also been a result of random jitter in echolocation (see Additional file 7: Figure S6).

Next, we examined whether the bats memorized their new-learned echolocation strategy in the constantly cluttered environment, adding it to their repertoire of sensing strategies, or whether they would have to re-learn it. Thus, after flying in the large flight room for *6 months*, each bat was placed again individually in the constantly cluttered flight chamber (stage 4). The bats used shorter IGIs immediately after returning to the cluttered environment, proving that they integrated the adapted echolocation strategy into their sensing repertoire (Fig. 2C, D). We compared the IGI of the last two weeks of the bats’ first encounter with the cluttered chamber with the first 2 weeks of the second encounter and found no significant difference between the two (one-way repeated measures Anova, *p* = 0.98, *F* = 15.3, *n* = 5 bats; for individual bats see Additional file 4: Table S1). Bat 5, which was the only bat that did not decrease IGI during the 2 months, also did not show evidence for integrating its learning, suggesting that this bat was using a different strategy.

To further validate that the bats adapted their sensing to the new type of environment (i.e., the degree of clutter) and not to a specific environment, two of the bats were additionally placed in *a different* environment with a similar degree of clutter (6 months after the first encounter). This was achieved by adding multiple new reflectors to the cluttered chamber and thus substantially changing the acoustic scene the bats experienced in comparison to their previous encounter with the chamber (see the “Methods” section). These two bats also immediately used the short IGI values in this novel cluttered environment, suggesting that they learned a general sensing strategy rather than a specific environment (Additional file 3: Figure S3).

In the case of pulse intensity that showed a change throughout the 2 months, there was no clear consistent response when comparing the first encounter to the second encounter (Additional file 5: Figure S4C).

## Discussion

Bats’ ability to move between different environments, often within seconds, dictates their need for sensory flexibility. Numerous studies have shown bats’ ability to adjust their echolocation rapidly [16–26]. Here we show that bats not only adapt their echolocation parameters in accordance with environmental changes within milliseconds, but that they are able to adapt their echolocation strategy over long time periods when encountering a new environment, and that they will incorporate this new adaptation into their sensing repertoire. Our results suggest that in theory, the ability to make long-term adaptations in addition to immediate ones could allow some bats to expand or change the range of habitats they forage in while adapting their echolocation accordingly over time (see below). We have shown that bats can decrease inter-pulse intervals when transferred into a cluttered environment, but the opposite might also be possible, that is, increasing intervals when transferred to an environment more open than previously encountered.

Taking into account the living habitat of *P. kuhlii* bats and their typical behavior, it is probably safe to assume that they have never experienced an environment as cluttered as we placed them in for such a long duration. It took the bats between 2 and 61 days until they started landing in the new environment. It seems that during this period the bats had to learn the motor task of taking off from one end of the chamber and landing on the target. When first placed in the cluttered chamber, the bats either attempted to reach the food plate from the side walls at the nearest point to the platform, or from the floor beneath it. Since these areas were shielded with nylon wires, they eventually learned to fly directly from the roost wall. Early attempts resulted in either landing on the floor or turning around before reaching the platform, and as a result crashing into the side walls. Eventually, all bats landed successfully and continued to do so from that point on. The new environment made the task difficult for several reasons: (1) The landing platform was relatively close, located 140 cm from the roost wall (Fig. 2A), making the motor task challenging. (2) The small dimensions of the chamber resulted in echoes returning to the bat from the surrounding walls prior to the target echoes [27], making the task sensory-challenging. (3) The task was also challenging from a neural point of view. Past work shows that the distribution of the best tuning of delay tuned neurons depends of the environment experienced by the animal [34] and is adaptive in accordance with it. Our bats which were used to a large flight room might have not sufficiently encoded such short distances in their brain. The distribution of best delays might have changed over time or alternatively, and as already suggested for a similar system [27], it is possible that some form of behavioral overriding of neuronal processes occurred, enabling the bats to eventually perform the task successfully. All of these challenges suggest that this short flight to landing requires a fine-tuned sensorimotor sequence of actions tightly timed to incoming input. They might explain why it took some bats a long time to learn the task.

Once landing was initiated and over the course of 2 months, the bats gradually decreased the pulse intervals before take-off (the IGI). Interestingly, they did not decrease the IGI before landing for the first time so this adaptation was not necessary for completing the task (Additional file 8: Figure S7). Moreover, they did not increase the number of pulses in the flight sequence, suggesting that the purpose of this adaptation was not to increase information flow. What then was the function of lowering the IGI over-time? In light of the high temporal precision required from the sensorimotor approach sequence [27], we hypothesize that this sequence should start with an accurate interval, which times the entire approach, similar to how a high-jumper counts steps backwards from the bar to the starting point in order to start its approach with the right step. Reducing the pre-takeoff interval probably makes the transition from stationary to in-flight echolocation smoother as can be learned from the sequences in Fig. 2B. This first interval before take-off times the entire approach sequence, so we hypothesize that it might serve to coordinate the entire sensorimotor sequence, similar to how the percussionist determines the rhythm of a melody with the first beat [35, 36]. We hypothesize that bats reduced the IGI over-time to adapt it to the highly increased clutter they experienced. This notion is also supported by our previous study, which showed that the last IGI correlates with the range of the target. This study also revealed that bats did not change their flight speed over time suggesting that our finding was related to sensory adaptation. Together with our previous study [27], the current study strengthens the importance of sensory planning before initiation of an approach.

But why was the decrease so gradual and not immediate upon the initiation of the first landing? It is known that adult animals not only have less plasticity than young animals but often do not respond to large scale changes at all [9, 37]. While in young animals, environmental changes inflict rapid neural plasticity; in adults, such pathways often consolidate in order to maintain stability. Several studies looking at the ability of barn owls to correct localization after manipulation in the visual field found that juveniles were able to adapt after a large-scale prism manipulation whereas adults were not [38, 39]. It is possible that if juveniles were tested in the same setup, they would have adjusted much faster to the new environment. Similar gradual learning has been shown for adult Barn owls after incremental training [9]. In a recent study, we showed that the tight sensorimotor approach sequence is innately encoded in new-born bats [40], but here we suggest that its initiation can be adapted over time even in adults. The adaptation reported here might have originated in the frontal cortex of the bat’s brain. This area is known to play a part in working memory and exhibits long-term plasticity and has been reported to be involved in both vocal motor and auditory processing in several bat species [41–45].

Note that bat 5 showed reversed behavior throughout the experiment. This bat did not show the same pattern of sensory adjustments and in fact was using short IGIs and a higher pulse rate already at the beginning of the experiment. One might hypothesize that this bat had previous experience with such cluttered environments, but it is impossible to conclude the reason for its unusual pattern.

Lastly, we found that on return to the constantly cluttered environment, many months later, the bats immediately used the previously learned extended echolocation strategy, demonstrating long-term learning of improved sensory acquisition in adult animals.

## Conclusions

Little is known about animals’ abilities to adapt their sensing during life. In this study, we demonstrate long-term plasticity in sensory acquisition in adult bats. Our results indicate that adaptive learning over long-time periods can enhance sensory planning before initiation of an approach when adapting to a new environment. Notably, echolocation allows measuring subtle changes in sensing, making it an advantageous system to study, but we predict that similar plasticity would be found in other active sensing systems such as rodent whisking and human eye movements.

## Methods

### Animals

Five *Pipistrellus kuhlii* bats were captured under permit from the Israeli National Park Authority (permit number 2016/41421). Bats were housed at Tel Aviv university’s Zoological gardens in a reversed light-dark cycle at a temperature of 23–26 °C. Experimental protocols and procedures were approved and performed according to the Institutional Animal Care and Use Committee operating according to the Israel Health Ministry (ethics approval number: 04-18-026).

### Experimental setup

The experiment consisted of two different environments: (1) a large 5.5 × 4.5 × 2.5 m^3^ flight room with acoustic foam on the walls and ceiling served as the less cluttered environment (Fig. 1B). In this room, bats were trained to land on a 110-cm high platform in the center of the room where mealworms were offered. (2) A smaller elongated 200 × 50 × 50 cm^3^ flight chamber that had acoustic foam on the walls and floor and a transparent acrylic ceiling served as a constantly cluttered environment. In this chamber, a 25-cm high landing platform was placed 140 cm from a wooden roost that was on the opposite wall. The remaining three walls of the chamber were shielded with nylon wire, forcing the bats to fly from the un-shielded roost (Fig. 2A). Only landings that originated from the roost wall were analyzed. The bats had to learn to fly from the roost to the landing platform in order to get to the food (a small metal plate with mealworms was present at all time). This task took between 2 and 61 days for different bats to learn. Up until then, to ensure feeding, mealworms were also offered at the base of the platform in smaller amounts throughout the week. This motivated the bats to fly to the platform even if they did not manage to land on it yet.

### Experimental design

In the first stage of the experiment, the bats resided in a 30 × 30 × 40 cm roost in a separate room and were released into the large flight room once a day. These sessions took place 5 days a week for at least 1 month, at which point a baseline was recorded over a 3-day period (during baseline recordings, each bat flew individually). In the second stage, the bats were transferred, in pairs, into the smaller cluttered flight chamber, where they stayed for 2 months (which started once each bat began landing). This phase was conducted for each pair in the same chamber in consecutive sessions, that is, we had one chamber so the entire experimental cycle was performed for pairs of bats sequentially. The individual bats within the pairs never started landing at the same time (there was a gap of 40 ± 15.5 days (mean ± SD) for the onset of landing of the two bats). Bats would fly to the landing platform to eat throughout the dark phase of their day and they slept in the roost of the chamber during the light phase (see Additional file 9: Table S2 for individual landing information). In the third stage, the bats were returned to the large flight room and recorded for 4–8 days (spread across 2 weeks, stage 3). They continued to fly in this environment (in the same conditions as the first stage) for 6 months. After 6 months, the bats were again moved into the smaller flight chamber, one at a time, and remained there for 2 weeks (stage 4). In this fourth and final stage, ten reflectors (made out of tin foil) were placed along the flight chamber—four on each side and two on the back wall (Additional file 1: Figure S1), to add acoustic complexity to the environment (for two out of the five bats). The other three bats were returned to the smaller chamber without the reflectors but were recorded again, with the reflectors too, 6 months after the end of this stage. This additional condition aimed to validate that the bats adjusted to new degree of clutter and not to a specific environment. In all, the experiment was comprised of four stages (Additional file 1: Figure S1): 1) 1 to 6 months in the less cluttered environment, (2) 2 months in the constantly cluttered environment, (3) 6 months in the less cluttered environment, and (4) 2 weeks in the constantly cluttered (same or enhanced) environment.

### Tracking, video, and audio recordings

In the large flight room, tracking was performed with a Motion Analysis Corp system. Twenty cameras (16 Raptor cameras, 1280 × 1024 pixels and 4 Raptor 12 cameras, 4096 × 3072 pixels) were used to track the bats at a frame rate of 200 fps. Two spherical reflectors (2.4-mm diameter, 3X3 Designs Corp.) were attached to the bats using double-sided tape. One reflector was mounted between the shoulder blades and the other was placed on the wing. Previous experiments confirmed that this system was able to track a moving reflector with an accuracy of ~1mm [46]. Audio recordings were performed with an ultrasonic wide-band microphone placed on the landing target (USG Electret Ultrasound Microphones - Avisoft Bioacoustics / Knowles FG) connected to a Hm1216 AD converter sampling at a rate of 375,000 Hz. Audio recordings were synchronized to the video tracking (Motion Analysis, Inc). In the smaller flight chamber, audio and video were recorded throughout the dark phase. Video was recorded with two IR-sensitive surveillance cameras. Audio was recorded using two electret ultrasonic microphones (Avisoft-Bioacoustics Knowles FG-O), connected to Hm1216 AD converter sampling at a rate of 250,000 Hz. One microphone was positioned in front of the roost and the other at the back of the chamber, behind the platform, facing the direction of flight. Both video and audio recordings were triggered automatically by either motion detection or the audio intensity threshold respectively.

### Audio analysis

Echolocation signal parameter extraction was performed in Batalef, a Matlab-based in-house software created for acoustic analysis [47, 48]. For each landing event, the echolocation sequence was analyzed. For recordings made in the smaller flight chamber, we extracted four parameters from the last pulse before takeoff (Fig. 2B): signal duration (defined according to a decrease of −12 dB relative to the peak), peak frequency (frequency with most energy), peak intensity, and inter-group-interval (IGI—defined as the time between the start of the last pulse before takeoff and the first pulse after takeoff). The analysis of stage 2 (2 months in the cluttered flight chamber) was done for every other day over 2 months. Days that had fewer than two landings were binned with the consecutive day and bats that had less recorded days (due to equipment failure) had additional days analyzed, resulting in all bats having 30 days of analyzed data. For the large flight room, pulse analysis was performed on the entire sequence of the last 150 cm before landing (approach phase). Parameters were measured from the envelope of the time signal.

### Statistics

To test the change in echolocation signal parameters over time in the small flight chamber, a Pearson linear regression test was performed for each individual and a generalized mixed-effect linear model (least squares) was performed for the group with the echolocation parameter set as the explained factor, time (in day bins) set as a fixed factor, and bat ID as a random effect. Because we tested five different echolocation parameters, we used a Bonferroni correction (GLM *p*-values were multiplied by 5). The analysis was performed on the average of each parameter in each time bin (see the “Audio analysis” section). To test for changes in echolocation parameters over distance from the landing platform, in baseline recordings of the different stages in the large flight room (stages 1 and 3), we performed a repeated measures ANCOVA (with bat ID as random effect and distance as a covariate), and we then performed a Tukey HSD post hoc. Finally, to test for differences in parameters in the small flight chamber in different stages we ran a one-way ANOVA, followed by Tukey HSD post hoc. All analyses were performed in JMP software (SAS INSTITUTE Inc., USA).

## Supplementary Information

**Additional file 1: Figure S1.** Experimental design. The experiment consisted of four stages: stage 1: 1-6 months in the large flight room; stage 2: two months in the smaller flight chamber (first clutter encounter); stage 3: six months in the large flight room and stage 4: two weeks in the smaller flight chamber (same or enhanced; second clutter encounter). Three bats that went back into the same small flight chamber at stage 4 did an additional six months in the large flight room and then were moved into the enhanced flight chamber for two weeks. The enhanced chamber had ten tin foil reflectors added to the walls - four on each side and two on the back wall.

**Additional file 2: Figure S2.** Change in inter-pulse-interval (IPI) of individual bats in the large flight room. The IPI was measured in the last 150 cm of flight until landing (mean ± SE). Baselines were measured before the cluttered chamber experiment (stage 1; Blue), and immediately after spending two months in the cluttered flight chamber (stage 3; Red).

**Additional file 3: Figure S3.** Change in inter-group-interval (IGI) over time for individual bats in the small flight chamber. The IGI immediately before takeoff was measured every other day over two months in the first clutter encounter (brown line - linear fit, points show mean ± SE). Four out of five bats show a decrease in IGI over time. After six months in the large flight room the bats were returned to the small chamber and the IGI was measured again over two weeks (second encounter - pink (same) or blue (enhanced) circle). At this stage the bats used lower IGIs than those used in their first encounter with this environment. Bat 1 and bat 3 were re-tested in the enhanced small flight chamber after an additional six months (blue circles) and showed similar results to those recorded in the original chamber in the first encounter.

**Additional file 4: Table S1.***P*-values of individual bats. P values of the different statistical tests for the individual bats. We used Tukey HSD post hoc to correct for multiple comparisons within tests when necessary. Red values indicate significance below 0.05. Where arrows are depicted they indicate the direction of change. Bats 1 and 3 were re-tested in the enhanced chamber after an additional six months and showed similar results to those recorded in stage 4 (the same chamber, after the initial six months). Bat 2 did not have enough data in this stage due to very few landings. Notice that bat 3 had a significant increase in intensity and duration along the two months in the chamber. This bat gave birth to twins during the stay in the small flight chamber as did the additional bat staying in this chamber at the same time (the additional bat never landed on the platform and so was excluded from the experiment). The situation were four pups shared this chamber with the adult bat probably influenced both intensity and duration of pulses and possibly the increase in the number of pulses emitted during flight (although we see no interference with IGI). Mean change values were estimated by subtracting the mean value of the last two weeks from the mean value of the first two weeks in the chamber.

**Additional file 5: Figure S4.** Additional acoustic parameters in the small flight chamber. **(A)** There was a significant decrease in pulse intensity throughout the two months at the group level, however, this was not consistent at the individual level, see below (linear fit, points show mean ± SE). **(B)** pulse peak frequency did not change significantly throughout the two months for the group (linear fit, points show mean ± SE), however one individual showed a significant change in frequency. n = 5 for both graphs. Data was normalized by dividing each bat’s data points by the maximum value. **(C)** Intensity values of the five individual bats at three different time points along the experiment: the first two weeks in the chamber (beginning of cluttered phase), the last two weeks in the chamber (end of cluttered phase) and two weeks in the chamber after six months in the large flight room (2^nd^ encounter). There was no clear consistency in direction of change for different bats (mean ± SE). **(D)** Peak frequency values of the five individual bats at three different time points along the experiment. There was no clear consistency in direction of change for different bats (mean ± SE). Asterisk indicate a significant change in the same direction as the group.

**Additional file 6: Figure S5.** Change in pulse emission during flight in the small flight chamber. **(A)** The change in the number of emitted pulses during flight over two months for all bats (mean ± SE, n = 5). The initial decrease is a result of bat 5’s contribution. Data was normalized by the maximum value of each bat. **(B)** Change in the number of pulses emitted by individual bats between the start and end of the cluttered phase (mean ± SE). Asterisk indicate a significant change.

**Additional file 7: Figure S6.** Change in inter-pulse-interval (IPI) of control bats in the large flight room. In order to examine whether the change in IPI observed for some of the bats in the large flight room resulted from the time spent in high clutter, or was just random drift, we also recorded three control bats over two months in the large flight room (without spending time in the cluttered chamber). The control bats also showed jitter in their echolocation, which was sometimes significant (repeated measures ANCOVA, P = 0.23, F = 1.5, df = 1, n = 3 bats), suggesting that the changes we observed were probably a result of natural echolocation jitter. The IPI was measured in the last 150cm of flight until landing (mean ± SE). Baselines were measured at the beginning of the experiment (blue) and again after two months (red).

**Additional file 8: Figure S7.** Inter-group-interval (IGI) before and after the initiation of landing in the small flight chamber. There was no significant difference in IGI measured in the week before landing was initiated (prior to ‘first landing’) and the first week of landing (t-test, *p* >0.3). Examples are shown for two of the bats.

**Additional file 9: Table S2.** Number of analyzed landings out of the total examined, in the small cluttered chamber. Data is given for the first clutter encounter - the initial two months in this chamber (stage 2). The total number of landings accounts for landings that took place on analyzed days only (30 days for each bat). Within those days some landings could not be analyzed due to technical difficulties such as invalid audio files.

## Data Availability

The datasets generated and analyzed during the current study are available on Mendeley Data: 10.17632/wccbjdrrsg.1 [49].
