# Supplementary material for: Adaptive learning and recall of motor-sensory sequences in adult echolocating bats
Source: BMC Biol. 2021 Aug 19;19:164. doi: 10.1186/s12915-021-01099-w (PMC8377959; doi:10.1186/s12915-021-01099-w)
Supplement: Supplementary file 1 — Additional file 1: Figure S1. Experimental design. The experiment consisted of four stages: stage 1: 1-6 months in the large flight room; stage 2: two months in the smaller flight chamber (first clutter encounter); stage 3: six months in the large flight room and stage 4: two weeks in the smaller flight chamber (same or enhanced; second clutter encounter). Three bats that went back into the same small flight chamber at stage 4 did an additional six months in the large flight room and then were moved into the enhanced flight chamber for two weeks. The enhanced chamber had ten tin foil reflectors added to the walls - four on each side and two on the back wall. [file 12915_2021_1099_MOESM1_ESM.pdf]

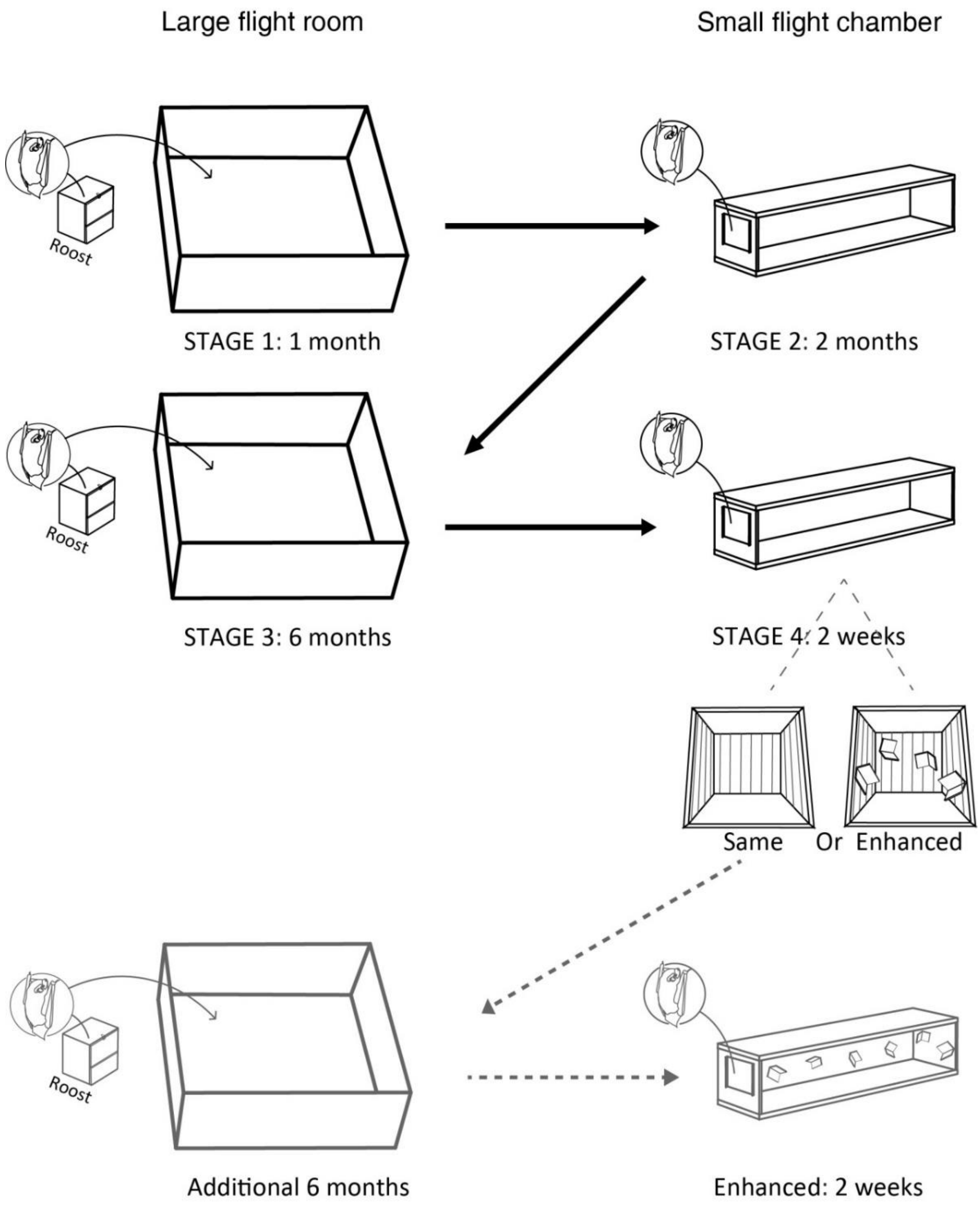

Figure S1: Experimental design. The experiment consisted of four stages: stage 1: 1-6 months in the large flight room; stage 2: two months in the smaller flight chamber (first clutter encounter); stage 3: six months in the large flight room and stage 4: two weeks in the smaller flight chamber (same or enhanced; second clutter encounter). Three bats that went back into the same small flight chamber at stage 4 did an additional six months in the large flight room and then were moved into the enhanced flight chamber for two weeks. The enhanced chamber had ten tin foil reflectors added to the walls - four on each side and two on the back wall.
