# Supplementary material for: Adaptive learning and recall of motor-sensory sequences in adult echolocating bats
Source: BMC Biol. 2021 Aug 19;19:164. doi: 10.1186/s12915-021-01099-w (PMC8377959; doi:10.1186/s12915-021-01099-w)
Supplement: Supplementary file 2 — Additional file 2: Figure S2. Change in inter-pulse-interval (IPI) of individual bats in the large flight room. The IPI was measured in the last 150 cm of flight until landing (mean ± SE). Baselines were measured before the cluttered chamber experiment (stage 1; Blue), and immediately after spending two months in the cluttered flight chamber (stage 3; Red). [file 12915_2021_1099_MOESM2_ESM.pdf]

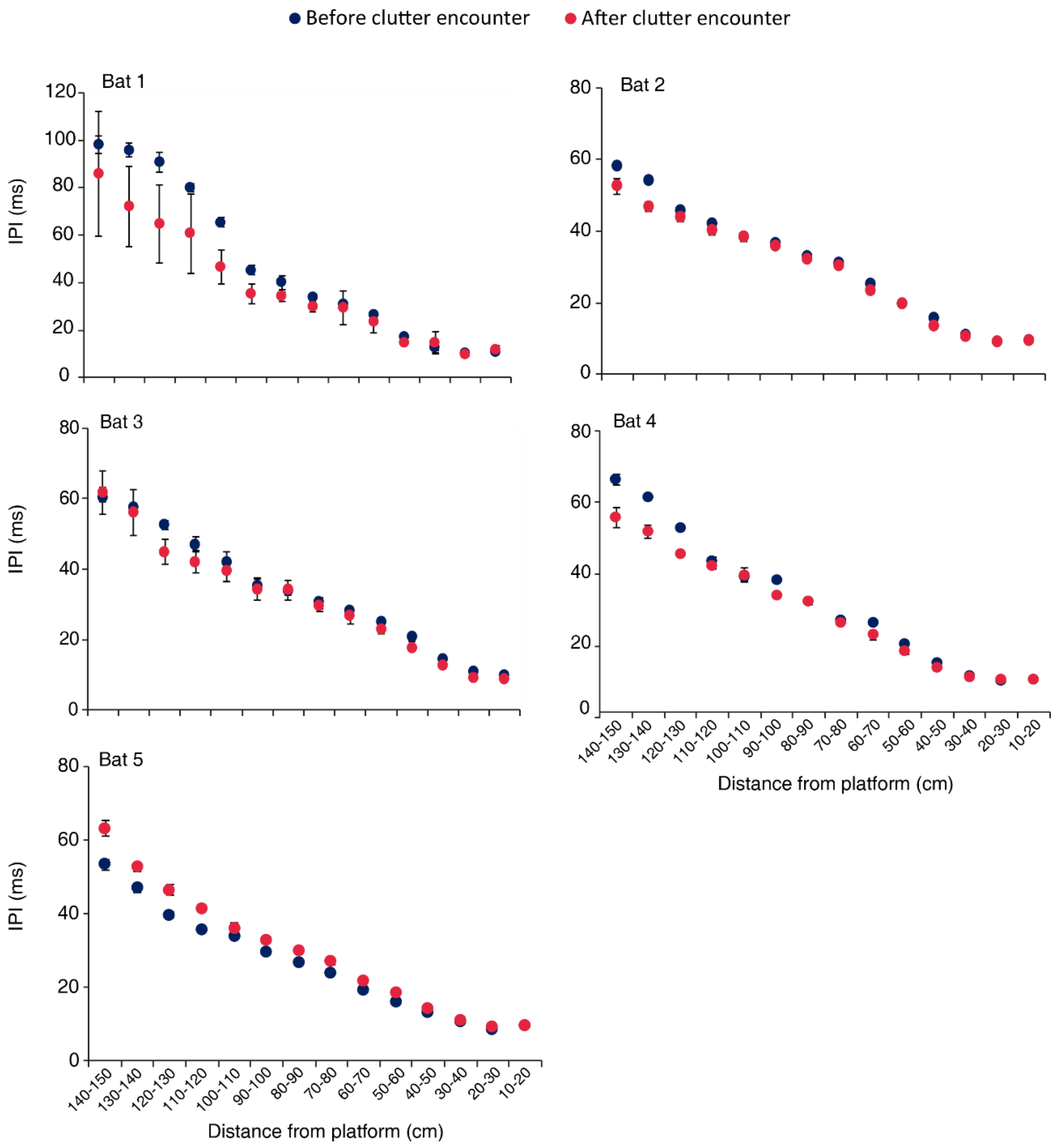

Figure S2: Change in inter-pulse-interval (IPI) of individual bats in the large flight room. The IPI was measured in the last 150cm of flight until landing (mean±SE). Baselines were measured before the cluttered chamber experiment (stage 1; Blue), and immediately after spending two months in the cluttered flight chamber (stage 3; Red).
