# Supplementary material for: Adaptive learning and recall of motor-sensory sequences in adult echolocating bats
Source: BMC Biol. 2021 Aug 19;19:164. doi: 10.1186/s12915-021-01099-w (PMC8377959; doi:10.1186/s12915-021-01099-w)
Supplement: Supplementary file 3 — Additional file 3: Figure S3. Change in inter-group-interval (IGI) over time for individual bats in the small flight chamber. The IGI immediately before takeoff was measured every other day over two months in the first clutter encounter (brown line - linear fit, points show mean ± SE). Four out of five bats show a decrease in IGI over time. After six months in the large flight room the bats were returned to the small chamber and the IGI was measured again over two weeks (second encounter - pink (same) or blue (enhanced) circle). At this stage the bats used lower IGIs than those used in their first encounter with this environment. Bat 1 and bat 3 were re-tested in the enhanced small flight chamber after an additional six months (blue circles) and showed similar results to those recorded in the original chamber in the first encounter. [file 12915_2021_1099_MOESM3_ESM.pdf]

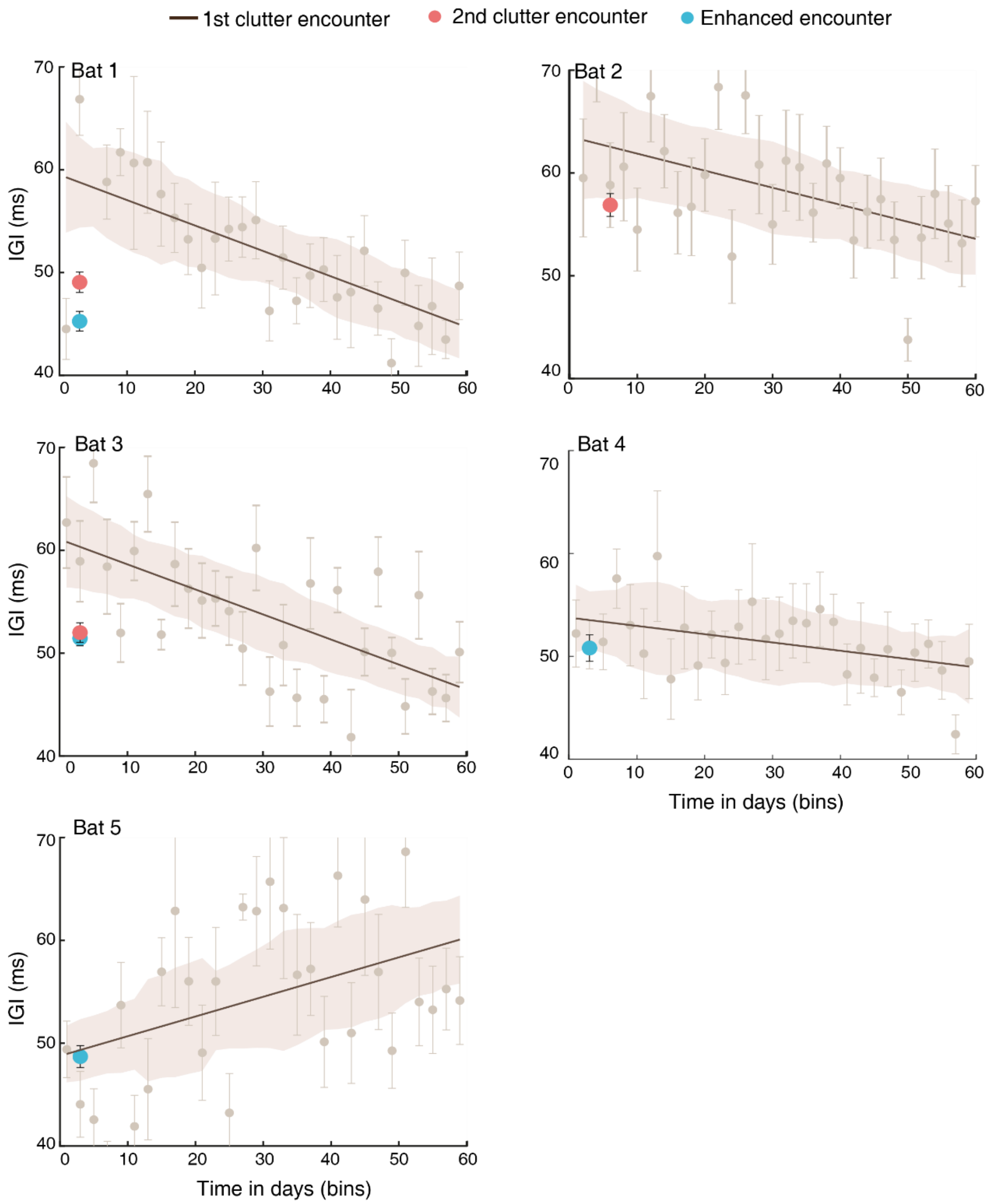

Figure S3: Change in inter-group-interval (IGI) over time for individual bats in the small flight chamber. The IGI immediately before takeoff was measured every other day over two months in the first clutter encounter (brown line - linear fit, points show mean±SE). Four out of five bats show a decrease in IGI over time. After six months in the large flight room the bats were returned to the small chamber and the IGI was measured again over two weeks (second encounter - pink (same) or blue (enhanced) circle). At this stage the bats used lower IGIs than those used in their first encounter with this environment. Bat 1 and bat 3 were re-tested in the enhanced small flight chamber after an additional six months (blue circles) and showed similar results to those recorded in the original chamber in the first encounter.
