# Supplementary material for: Adaptive learning and recall of motor-sensory sequences in adult echolocating bats
Source: BMC Biol. 2021 Aug 19;19:164. doi: 10.1186/s12915-021-01099-w (PMC8377959; doi:10.1186/s12915-021-01099-w)
Supplement: Supplementary file 4 — Additional file 4: Table S1.P-values of individual bats. P values of the different statistical tests for the individual bats. We used Tukey HSD post hoc to correct for multiple comparisons within tests when necessary. Red values indicate significance below 0.05. Where arrows are depicted they indicate the direction of change. Bats 1 and 3 were re-tested in the enhanced chamber after an additional six months and showed similar results to those recorded in stage 4 (the same chamber, after the initial six months). Bat 2 did not have enough data in this stage due to very few landings. Notice that bat 3 had a significant increase in intensity and duration along the two months in the chamber. This bat gave birth to twins during the stay in the small flight chamber as did the additional bat staying in this chamber at the same time (the additional bat never landed on the platform and so was excluded from the experiment). The situation were four pups shared this chamber with the adult bat probably influenced both intensity and duration of pulses and possibly the increase in the number of pulses emitted during flight (although we see no interference with IGI). Mean change values were estimated by subtracting the mean value of the last two weeks from the mean value of the first two weeks in the chamber. [file 12915_2021_1099_MOESM4_ESM.pdf]

Parameter	Test	Bat1	Bat2	Bat3	Bat4	Bat5
IGI	Linear regression along 2 months in the cluttered chamber	<0.0001↓	0.004↓	<0.0001↓	0.008↓	0.012↑
IGI	ANOVA (first 2 weeks vs. Last 2 weeks in the cluttered chamber)	<0.0001↓	0.0053↓	<0.0001↓	0.04↓	0.0005↑
IGI	ANOVA (first 2 weeks in the clutter vs. Stage4)	0.0001↓	0.037↓	0.0002↓	0.25	0.23
IGI	ANOVA (Last 2 weeks in the clutter vs. Stage4)	0.64	0.39	0.67	0.38	0.006↓
IGI	ANCOVA (stage1 vs. stage3)	0.003↓	0.018↓	0.17	0.0025↓	<0.0001↑
IGI	ANOVA cluttered chamber after 6 months (same vs. enhanced)	0.13	NA	0.98	NA	NA
Number of emitted pulses in flight	Linear regression along 2 months in the cluttered chamber	0.0495↑	0.07	0.0005↑	0.66	<0.0001↓
Intensity	Linear regression along 2 months in the cluttered chamber	0.2	0.2	0.0001↑	<0.0001↓	0.005↓
Peak frequency	Linear regression along 2 months in the cluttered chamber	0.46	0.051	0.65	0.07	0.0004↑
Duration	Linear regression along 2 months in the cluttered chamber	0.8	0.7	0.006↑	0.58	0.004↓
IGI	Mean change (ms)	13.77↓	8.3↓	9.5↓	5.15↓	10.8↑
Intensity	Mean change (dB)	0.99↓	0.67↓	4.27↑	6.00↓	1.75↓
Peak frequency	Mean change (Hz)	51.72↑	1662.71↑	460.28↓	773.92↓	2264.81↑
Duration	Mean change (ms)	0.03↑	0.01↑	0.28↑	0.02↓	0.10↓
Number of emitted pulses in flight	Mean change (count)	0.035↑	1.5↑	4.1↑	0.35↓	21.3↓

Table S1: P-values of individual bats. P values of the different statistical tests for the individual bats. We used Tukey HSD post hoc to correct for multiple comparisons within tests when necessary. Red values indicate significance below 0.05. Where arrows are depicted they indicate the direction of change. Bats 1 and 3 were re-tested in the enhanced acoustic environment after an additional six months and showed similar results to those recorded in stage 4 (the same chamber, after the initial six months). Bat 2 did not have enough data in this stage due to very few landings. Notice that bat 3 had a significant increase in intensity and duration along the two months in the chamber. This bat gave birth to twins during the stay in the small flight chamber as did the additional bat staying in this chamber at the same time (the additional bat never landed on the platform and so was excluded from the experiment). The situation were four pups shared this chamber with the adult bat probably influenced both intensity and duration of pulses and possibly the increase in the number of pulses emitted during flight (although we see no interference with IGI). Mean change values were estimated by subtracting the mean value of the last two weeks from the mean value of the first two weeks in the chamber.
