# Supplementary material for: Adaptive learning and recall of motor-sensory sequences in adult echolocating bats
Source: BMC Biol. 2021 Aug 19;19:164. doi: 10.1186/s12915-021-01099-w (PMC8377959; doi:10.1186/s12915-021-01099-w)
Supplement: Supplementary file 5 — Additional file 5: Figure S4. Additional acoustic parameters in the small flight chamber. (A) There was a significant decrease in pulse intensity throughout the two months at the group level, however, this was not consistent at the individual level, see below (linear fit, points show mean ± SE). (B) pulse peak frequency did not change significantly throughout the two months for the group (linear fit, points show mean ± SE), however one individual showed a significant change in frequency. n = 5 for both graphs. Data was normalized by dividing each bat’s data points by the maximum value. (C) Intensity values of the five individual bats at three different time points along the experiment: the first two weeks in the chamber (beginning of cluttered phase), the last two weeks in the chamber (end of cluttered phase) and two weeks in the chamber after six months in the large flight room (2nd encounter). There was no clear consistency in direction of change for different bats (mean ± SE). (D) Peak frequency values of the five individual bats at three different time points along the experiment. There was no clear consistency in direction of change for different bats (mean ± SE). Asterisk indicate a significant change in the same direction as the group. [file 12915_2021_1099_MOESM5_ESM.pdf]

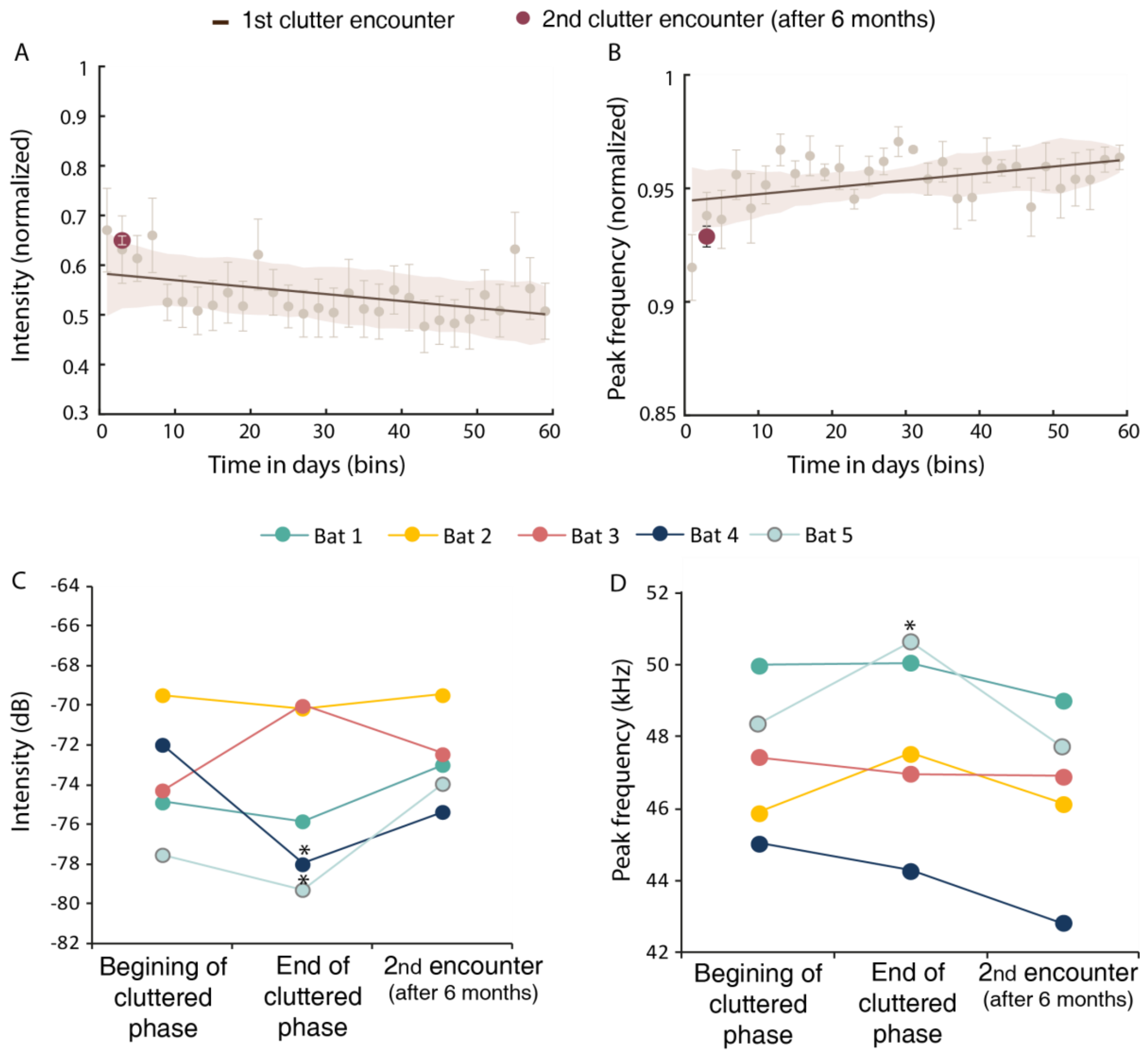

Figure S4: Additional acoustic parameters in the small flight chamber. (A) There was a significant decrease in pulse intensity throughout the two months at the group level, however, this was not consistent at the individual level, see below (linear fit, points show mean±SE). **(B)** Pulse peak frequency did not change significantly throughout the two months for the group (linear fit, points show mean±SE), however one individual showed a significant change in frequency. n=5 for both graphs. Data was normalized by dividing each bat's data points by the maximum value. **(C)** Intensity values of the five individual bats at three different time points along the experiment: the first two weeks in the chamber (beginning of cluttered phase), the last two weeks in the chamber (end of cluttered phase) and two weeks in the chamber after six months in the large flight room (2nd encounter). There was no clear consistency in direction of change for different bats (mean±SE). **(D)** Peak frequency values of the five individual bats at three different time points along the experiment. There was no clear consistency in direction of change for different bats (mean±SE). Asterisk indicate a significant change in the same direction as the group.
