# Supplementary material for: Adaptive learning and recall of motor-sensory sequences in adult echolocating bats
Source: BMC Biol. 2021 Aug 19;19:164. doi: 10.1186/s12915-021-01099-w (PMC8377959; doi:10.1186/s12915-021-01099-w)
Supplement: Supplementary file 6 — Additional file 6: Figure S5. Change in pulse emission during flight in the small flight chamber. (A) The change in the number of emitted pulses during flight over two months for all bats (mean ± SE, n = 5). The initial decrease is a result of bat 5’s contribution. Data was normalized by the maximum value of each bat. (B) Change in the number of pulses emitted by individual bats between the start and end of the cluttered phase (mean ± SE). Asterisk indicate a significant change. [file 12915_2021_1099_MOESM6_ESM.pdf]

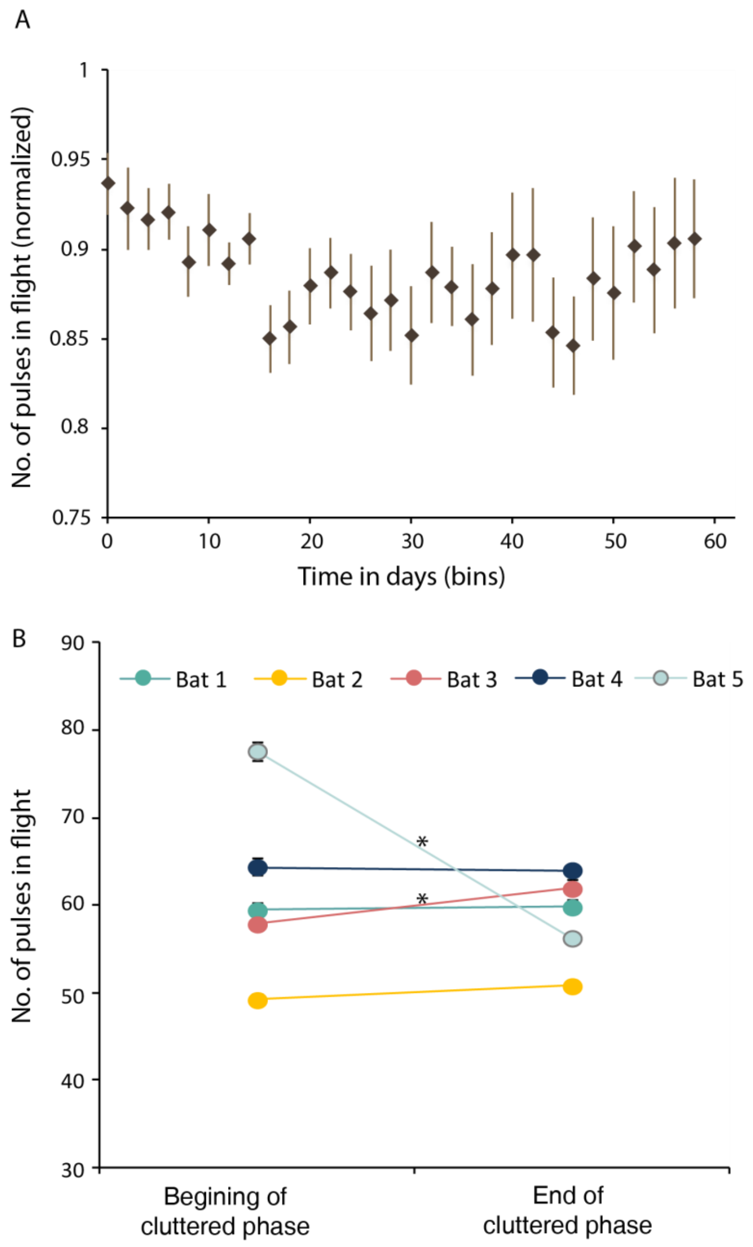

Figure S5: Change in pulse emission during flight in the small flight chamber. (A) The change in the number of emitted pulses during flight over two months for all bats (mean±SE, n=5). The initial decrease is a result of bat 5's contribution. Data was normalized by the maximum value of each bat. **(B)** Change in the number of pulses emitted by individual bats between the start and end of the cluttered phase (mean±SE). Asterisk indicate a significant change.
