# Supplementary material for: Adaptive learning and recall of motor-sensory sequences in adult echolocating bats
Source: BMC Biol. 2021 Aug 19;19:164. doi: 10.1186/s12915-021-01099-w (PMC8377959; doi:10.1186/s12915-021-01099-w)
Supplement: Supplementary file 7 — Additional file 7: Figure S6. Change in inter-pulse-interval (IPI) of control bats in the large flight room. In order to examine whether the change in IPI observed for some of the bats in the large flight room resulted from the time spent in high clutter, or was just random drift, we also recorded three control bats over two months in the large flight room (without spending time in the cluttered chamber). The control bats also showed jitter in their echolocation, which was sometimes significant (repeated measures ANCOVA, P = 0.23, F = 1.5, df = 1, n = 3 bats), suggesting that the changes we observed were probably a result of natural echolocation jitter. The IPI was measured in the last 150cm of flight until landing (mean ± SE). Baselines were measured at the beginning of the experiment (blue) and again after two months (red). [file 12915_2021_1099_MOESM7_ESM.pdf]

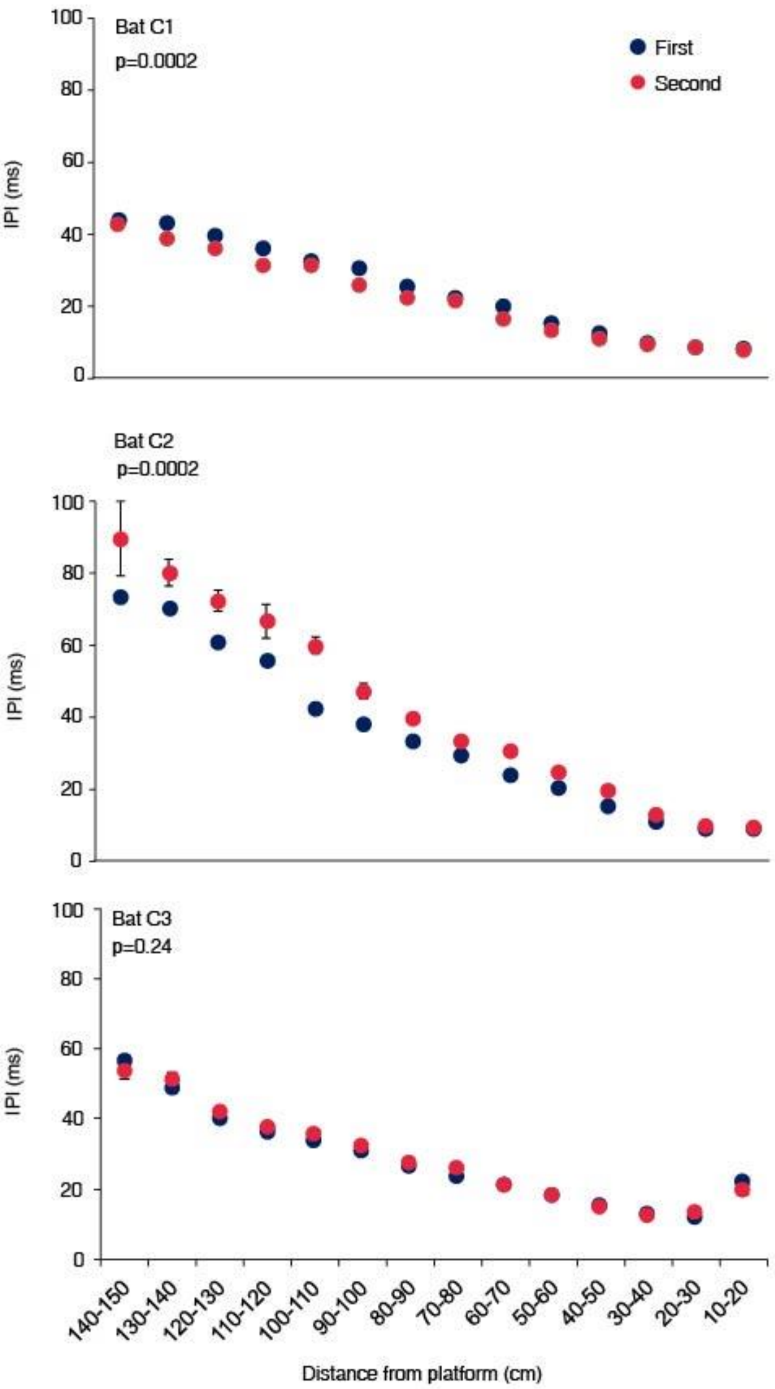

Figure S6: Change in inter-pulse-interval (IPI) of control bats in the large flight room. In order to examine whether the change in IPI observed for some of the bats in the large flight room resulted from the time spent in high clutter, or was just random drift, we also recorded three control bats over two months in the large flight room (without spending time in the cluttered chamber). The control bats also showed jitter in their echolocation, which was sometimes significant (repeated measures ANCOVA, P=0.23, F=1.5, df=1, n=3 bats), suggesting that the changes we observed were probably a result of natural echolocation jitter. The IPI was measured in the last 150cm of flight until landing (mean±SE). Baselines were measured at the beginning of the experiment (blue) and again after two months (red).
