# Supplementary material for: Adaptive learning and recall of motor-sensory sequences in adult echolocating bats
Source: BMC Biol. 2021 Aug 19;19:164. doi: 10.1186/s12915-021-01099-w (PMC8377959; doi:10.1186/s12915-021-01099-w)
Supplement: Supplementary file 8 — Additional file 8: Figure S7. Inter-group-interval (IGI) before and after the initiation of landing in the small flight chamber. There was no significant difference in IGI measured in the week before landing was initiated (prior to ‘first landing’) and the first week of landing (t-test, p >0.3). Examples are shown for two of the bats. [file 12915_2021_1099_MOESM8_ESM.pdf]

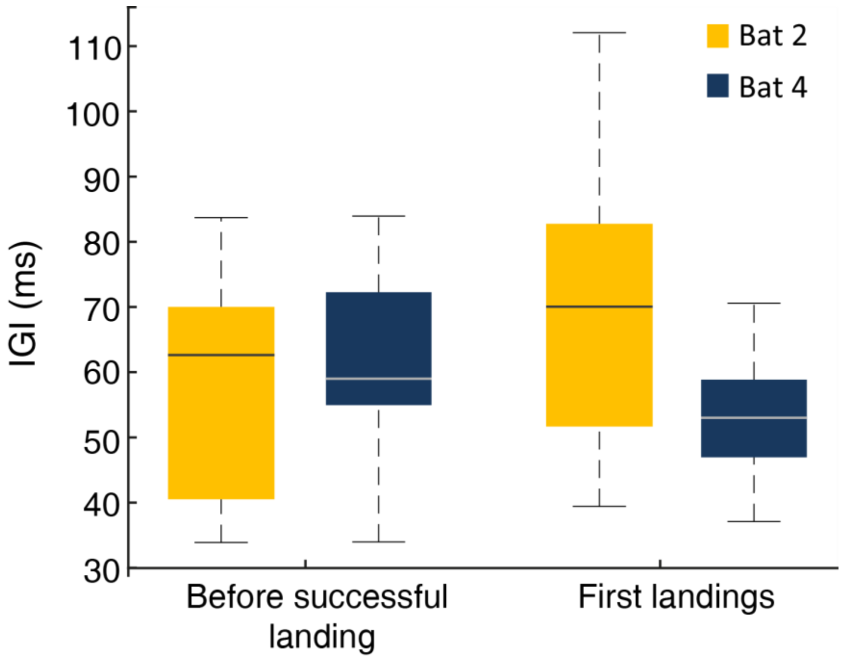

Figure S7: Inter-group-interval (IGI) before and after the initiation of landing in the small flight chamber. There was no significant difference in IGI measured in the week before landing was initiated (prior to 'first landing') and the first week of landing (t-test, p>0.3). Examples are shown for two of the bats.
