# Supplementary material for: Adaptive learning and recall of motor-sensory sequences in adult echolocating bats
Source: BMC Biol. 2021 Aug 19;19:164. doi: 10.1186/s12915-021-01099-w (PMC8377959; doi:10.1186/s12915-021-01099-w)
Supplement: Supplementary file 9 — Additional file 9: Table S2. Number of analyzed landings out of the total examined, in the small cluttered chamber. Data is given for the first clutter encounter - the initial two months in this chamber (stage 2). The total number of landings accounts for landings that took place on analyzed days only (30 days for each bat). Within those days some landings could not be analyzed due to technical difficulties such as invalid audio files. [file 12915_2021_1099_MOESM9_ESM.pdf]

	No. of landings analyzed	No. of total landings	Avg. landings per day (mean±SD)
Bat 1	329	410	12.8±5.3
Bat 2	383	472	15.2±5.2
Bat 3	294	409	11±8.8
Bat 4	388	389	12.1±5.3
Bat 5	282	284	8.3±5

Table S2: Number of analyzed landings out of the total examined, in the small cluttered chamber. Data is given for the first clutter encounter - the initial two months in this chamber (stage 2). The total number of landings accounts for landings that took place on analyzed days only (30 days for each bat). Within those days some landings could not be analyzed due to technical difficulties such as invalid audio files.
